# Exploring the Relationships between Posttraumatic Growth, Wisdom, and Quality of Life in Older Cancer Survivors

**DOI:** 10.31557/APJCP.2019.20.9.2667

**Published:** 2019

**Authors:** Seung-Kyoung Yang, Yeongmi Ha

**Affiliations:** 1 *Department of Nursing, Kyungnam University, Changwon, *; 2 *College of Nursing and Institute of Health Sciences, Gyeongsang National University, Jinju, South-Korea. *

**Keywords:** Posttraumatic growth, wisdom, quality of life, elderly, cancer survivor

## Abstract

**Objective:**

The number of older cancer survivors is steadily rising with a growing aging population, and a great interest in evaluating the quality of life is emerged. Although understanding how to improve the quality of life in older cancer survivors is critical as the number of older survivors continues to grow in communities, little is known about empirical evidence regarding predictors of the quality of life in older cancer survivors. This study aimed to examine relationships between posttraumatic growth, wisdom, and quality of life in older cancer survivors.

**Methods:**

A convenience sample of older cancer survivors after completing cancer treatments (n=121) participated from one public health center, and they filled out self-report questionnaires on measures of posttraumatic growth, wisdom, and quality of life.

**Results:**

As results of multiple regression analysis, the most significant factor on each domain of the quality of life has shown that higher levels of subjective economic status were associated with significant improvement of four domains of quality of life, and wisdom and posttraumatic growth were associated with significant improvement in social/family well-being.

**Conclusion:**

This study highlights predictors of each domain of quality of life that subjective economic status, posttraumatic growth and wisdom significantly affected the quality of life in older cancer survivors. Findings indicate that psychological interventions need to be developed and implemented for older cancer survivors to prevent long-term effects of cancer and to increase their quality of life. For improving their quality of life, primary care providers or community health professionals need to develop tailored interventions, such as home-based cancer survivorship programs.

## Introduction

Advances in early detection and new cancer treatments have led to an increase in cancer survivors. It is estimated that the 5-year cancer survival rate was 70.7% in Korea from 2011 to 2015 (National Cancer Information Center, 2018), and 66.9% in U.S. from 2008 to 2015 (National Cancer Institutes, 2018). With a growing number of elderly population and an improvement of cancer prognosis, cancer survivors being older than 65 years are also increasing (Rowland and Bellizzi, 2014). According to a study on cancer survivors and aging population, approximately 60% of cancer survivors are age 65 or older, many of whom have multiple health problems and various survivorship needs (Parry et al., 2011).

With increasing trends of cancer survivors, a growing interest in evaluating the quality of life (QoL) of cancer survivors is emerged (Thong et al., 2013). The period after completing cancer treatments is often described as more difficult than the treatment itself because long term effects of cancer treatments such as fatigue, depression, sleep disorders, functional disability, and pain, are highly prevalent in cancer survivors (Wu and Harden, 2015; Mayer et al., 2017). Such bothersome symptoms after cancer treatments continues to have a negative impact on overall quality of life in cancer survivors both physically and psychologically, and the QoL of cancer survivors is known to be poor (Cimprich et al., 2002; Stanton et al., 2005; Wu and Harden, 2015). 

Positive psychological change often occurs as a result of struggling with a traumatic event, which is called posttraumatic growth (PTG; Tedeschi and Calhoun, 2004). PTG has been viewed as a positive adaptation in response to a cancer and treatments that can give meaningful cognitive processing (Helgeson et al., 2006). During the survivorship period, some cancer survivors derive benefits and a more positive perspective toward life from their traumatic events by finding meaning in their experience of cancer diagnosis and by rebuilding meaningful interpersonal relationship. Although many research in the area of PTG have examined positive growth and psychological adaptation within cancer survivorship journey, findings on the association between PTG and psychological outcomes such as QoL and have found mixed or null relations between them (Stanton et al., 2005; Helgeson et al., 2006; Bellizzi et al., 2010; Sim et al., 2014). 

Wisdom is considered as psychosocial maturity that integrates cognitive, reflective, and emotional personality traits (Karelitz et al., 2010). The concept of wisdom often conjures the stereotypical image of the “old wise man” (Weststrate et al., 2016). Wisdom accumulates through life experiences, and it has been reported that age affects the depth of wisdom, and that life wisdom advances with aging (Ardelt, 2016). For example, a previous study has reported that the elderly, who have more diverse life experiences than do the young, are wiser (Ardelt, 2016). It has recently been indicated that wisdom may have significant relationships between individuals and health outcomes via improvements in physical health and psychological health such as resilience and QoL (Ardelt, 1997; Jeste and Oswald, 2014). Many cancer survivors describe the process of living with cancer as a life-changing experience. Even though older cancer survivors have more challenging life experience which might lead to better coping strategies and wisdom, little research has been conducted to specifically investigate how wisdom affects the QoL of older cancer survivors after completing cancer treatments. 

Despite the majority of cancer survivors being elderly, little is known about empirical evidence regarding older cancer survivors after completing cancer treatments. While the majority studies have been conducted with QoL of patients receiving active treatment, few studies have focused on community-dwelling older cancer survivors after completing cancer treatments. It is evident that older cancer survivors in a community continue to struggle with declined functional status, various physical symptoms and psychological issues. Although understanding how to improve the QoL of older cancer survivors is critical as the number of older survivors continues to grow in communities, little is known about empirical evidence regarding the predictors of QoL in older cancer survivors. Therefore, this study aimed to investigate relationships between PTG, wisdom, and QoL including physical, social/family, emotional, and functional well-being in community-dwelling cancer survivors after completing cancer treatments.

## Materials and Methods

*Participants*

The study was conducted on cancer survivors registered at a public health center in Korea, who resided in a community after the completion of all cancer treatments. The inclusion criteria were as follows: first, age of 50 or older; second, community dwelling cancer survivors after completing all cancer treatments; third, having abilities to communicate and sufficiently understand the survey. 

The sample size was estimated using the G*power 3.1.9.2 program. The criteria for linear multiple regression: statistical power (1-β) of .80, significance level (ɑ) = .05, medium effect size (f^2^) = .15, and eight predictors. It indicated that we needed a total of 109 participants. Allowing for attrition, we recruited a total of 150 people for the study. Questionnaires were distributed to 150 cancer survivors and returned entirely. Finally, a total of 121 questionnaires were analyzed after excluding 29 questionnaires due to missing data.

*Measurements*

Quality of life was assessed using the Functional Assessment Cancer Therapy-General (FACT-G) version 4 (Cella et al., 1993). The FACT-G is a cancer specific QoL measure containing a total of 27 items consisting of four subscales: physical well-being (7 items), social/family well-being (7 items), emotional well-being (6 items), functional well-being (7 items). All items are rated on a 5-point Likert scale ranging from 0(not at all) to 4(very much). Negatively worded items are reversely scored prior to summing so that higher subscale and total scores indicate better QoL. The Cronbach’s α was .87 in this study.

The Korean version of Posttraumatic Growth Inventory (Song et al., 2006) assessed the degree to which people who had experienced crisis events believe they have in some ways grown through experiencing the adversity. This scale has been designed to determine how successful individuals, coping with the aftermath of trauma, are in reconstructing or strengthening their perceptions of self, others, and the meaning of events. This scale includes 16 items consisting of four subscales: spiritual change (2 items), new possibilities (3 items), relating to others (5 items), changed perception of self (6 items). All items are rated on a 6-point Likert scale ranging from 0 (not at all) to 5 (very great degree). Higher score indicates a higher level of PTG. The Cronbach’s α was 0.80 in this study.

Wisdom was measured with the Self Wisdom Scale (Sung et al., 2010) for the Korean elderly. The scale includes 27-item consisting of three subscales: sympathetic emotion (11 items), self-reflection (9 items), and experience of overcoming hardships in life (7 items). The items are measured on 4-point Likert scale ranging from 1 (not at all) to 4 (very great degree). The Cronbach’s α was 0.70 in this study.

*Data collection*

Participants responded self-report questionnaires during February 2015. Some participants who could complete self-report questionnaires filled out survey questions on their own, and a researcher conducted face-to-face interview to complete a survey for elderly who could not complete it. 

*Ethical consideration*

The informed written consent was obtained from each participant before data collection. The purpose and procedures of the study were explained to all participants. Their participation was entirely voluntary and they were free to withdraw from the research at any time. Confidentiality was guaranteed to protect participants’ interests. 

*Data analysis*

All the analyses were conducted with SPSS software. First, the differences of each domain of QoL according to general and disease-related characteristics were analyzed using t-test or analysis of variance (ANOVA). Second, means and standard deviations of PTG, wisdom, and QoL were calculated. Third, Pearson’s correlations coefficient was conducted to examine the correlations between PTG, wisdom, and each domain of QoL. Fourth, multiple regression analysis was used to assess factors affecting on each domain of QoL of older cancer survivors.

## Results

*Differences of QoL subscales according to general and disease-related characteristics*

A general and disease-related characteristics of participants were shown in the Table 1. The mean age of the participants was 67.63±9.35, and 64.5% of the participants was women, and 56.2% of them was married. Approximately half of participants had completed less than a primary school. In the socioeconomic status (SES), 38.0% of participants answered ‘low’, and a majority of participants (95.0%) did not experience cancer recurrence.

The Differences in QoL subscales according to general and disease-related characteristics of the participants were shown in Table 1. Physical well-being was significantly different in SES (F=4.29, p=0.003) and cancer metastasis or recurrence (t=3.18, p=0.045). Social/family well-being was significantly different in marital status (t=3.99, p=0.000), education level (F=3.13, p=0.047), and SES (F=7.08, p=0.000). Emotional well-being was significantly different in age (F=3.27, p=0.042), gender (t=1.98, p=0.05), marital status (t=2.10, p=0.038), and SES (F=5.28, p=0.001). Lastly, functional well-being was significantly different in age (F=3.86, p=0.024), marital status (t=4.07, p=0.000), educational level (F=5.90, p=0.004), and SES (F=4.82, p=0.001). 

*Mean scores of PTG, wisdom and QoL*

With higher scores indicating greater perception of PTG, PTG scores ranged from 2.08 (spiritual change) to 2.77 (changed perception of self), indicating moderate PTG score (Table 2). Mean score for wisdom was 2.90 (SD=0.30, range 1-4), indicating moderate wisdom score. Mean score for each domain of QoL was 3.05 (physical well-being), 2.75 (emotional well-being), 2.25 (functional well-being), and 1.86 (social/family well-being), indicating moderate QoL for each subscale.

*Correlations between PTG, wisdom, and QoL*

The Pearson correlations of PTG, wisdom and QoL subscales were shown in Table 3. Physical well-being did not have a statistically significant correlation with PTG and wisdom. Social/family well-being had a significant positive correlation with PTG (r=0.45, p=0.000) and wisdom (r=0.42, p=0.000). Emotional well-being had a significant positive correlation with wisdom (r=0.21, p=0.024). Finally, functional well-being had a significant positive correlation with PTG (r=0.27, p=0.003) and wisdom (r=.40, p=.000).

*Predictors of QoL subscales*

As results of multiple regression analysis, it was found that SES (β=0.33, p <0.001) explained cancer survivors’ physical well-being with 13.6% (F = 6.14, p = 0.001) and emotional well-being with 19.5% (F = 4.60, p < 0.001). In social/family well-being, SES (β=0.28, p = 0.001), wisdom (β=0.20, p = 0.040), and PTG (β=0.24, p = 0.012) explained with 34.8% (F = 12.26, p < 0.001). And, it was found that SES (β=0.19, p = 0.035) and wisdom (β=0.29, p = 0.005) explained cancer survivors’ functional well-being with 26.0% (F = 6.66, p < 0.001) (Table 4).

## Discussion

Older cancer survivors often face negative physical, psychosocial, and functional consequences due to long-term effects of cancer treatments (Rawland and Bellizzi, 2014). It is well-known that their adverse physical and psychosocial functions influences on the QoL of cancer survivors. Our research has found that the mean score of physical well-being was the highest in older cancer survivors, and that of social well-being was the lowest. This finding is partially consistent with a study by Disipio et al., (2010) with breast cancer survivors has reported that physical well-being score was the highest, and emotional well-being score was the lowest. The reason why social well-being score is the lowest in this study is that half of older participants lived alone due to divorce or death of their spouse, and did not receive sufficient support from a spouse or their family, mainly because it would have been difficult for them to be involved in social life. Accordingly, formal and informal social support for older cancer survivors would be helpful in improving their QoL.

Interestingly, our study to identify determinants of four domains of the QoL has found that lower levels of SES in older cancer survivors negatively influenced all subdomains of the QoL including physical, social/family, emotional, and functional well-being. The literature consistently shows that differences in SES are associated with large disparities in multiple health outcomes including physical and psychological health status, disease prevalence, and quality of life (Regidor et al., 1999; Thumboo et al., 2003; Yen et al., 2006; Kim and Park, 2015). According to the 2014 Survey of Living Conditions and Welfare Needs of Korean Older Persons, 49.7% of the elderly perceived their SES was low, and 52.8% lived with a poverty-level income (Survey of Living Conditions and Welfare Needs of Korean Older Persons, 2014). More than a half of the elderly in Korea are socioeconomically vulnerable. In addition, cancer needs to be continuously managed even after treatment is complete, and thus, the accompanying economic burden may be a reason for the lower QoL.

**Table 1:** Differences of Each Domain of Quality of Life According to General and Disease Related Characteristics of the Participant (N=121)

Characteristic	n (%)	Physical well-being		Social/family well-being		Emotional well-being		Functional well-being	
		M (SD)	t or F (p)	M (SD)	t or F (p)	M (SD)	t or F(p)	M (SD)	t or F (p)
Age (years)									
50-59	34 (28.1)	2.83 (0.91)	1.93 (0.150)	1.89 (0.91	0.20 (0.820)	2.48 (0.91)	3.27 (0.042)	2.23 (0.94)	3.86 (0.024)
60-69	37 (30.6)	3.23 (0.83)		1.91 (0.89)		3.00 (0.71)		2.54 (0.61)	
70≤	50 (41.3)	3.06 (0.83)		1.80 (0.95)		2.74 (0.90)		2.05 (0.84)	
Gender									
Male	43 (35.5)	3.22 (0.76)	1.64 (0.105)	2.00 (0.85)	1.25 (0.213)	2.96 (0.84)	1.98 (0.050)	2.41 (0.86)	1.60 (0.112)
Female	78 (64.5)	2.95 (0.90)		1.50 (0.88)		2.63 (0.87)		2.16 (0.80)	
Marital status									
Married	68 (56.2)	3.16 (0.79)	1.70 (0.090)	2.14 (0.85)	3.99 (<0.001)	2.89 (0.80)	2.10 (0.038)	2.51 (0.78)	4.07 (<0.001)
Others	53 (43.8)	2.90 (0.92)		1.50 (0.88)		2.56 (0.92)		1.92 (0.78)	
Education									
≤Primary	55 (45.5)	3.04 (0.87)	0.06 (0.946)	1.71 (0.91)	3.13 (0.047)	2.80 (0.82)	2.94 (0.057)	2.10 (0.86)	5.90 (0.004)
Middle≤	52 (43.0)	3.03 (0.88)		1.88 (0.93)		2.57 (0.95)		2.23 (0.67)	
≥College	14 (11.6)	3.12 (0.82)		2.39 (0.74)		3.17 (0.49)		2.92 (0.98)	
Subjective economic status									
High	1 (0.8)	3.71 (0.00)	4.29 (0.003)	3.00 (0.00)	7.08 (<0.001)	3.66 (0.00)	5.28 (0.001)	2.57 (0.00)	4.82 (0.001)
Upper middle	5 (4.1)	3.25 (0.49)		2.89 (0.22)		3.56 (0.53)		3.34 (0.72)	
Middle	32 (26.4)	3.42 (0.58)		2.32 (0.79)		3.17 (0.60)		2.54 (0.73)	
Lower middle	37 (30.6)	3.13 (0.90)		1.74 (0.81)		2.57 (0.77)		2.17 (0.72)	
Low	46 (38.0)	2.68 (0.90)		1.50 (0.91)		2.40 (0.79)		1.99 (0.86)	
Cancer recurrence									
Yes	6 (5.0)	2.47 (1.26)	3.18 0 (0.045)	2.08 (1.13)	1.20 (0.304)	2.08 (1.21)	3.04 (0.051)	1.76 (1.21)	1.17 (0.315)
No	115 (95.0)	2.96 (0.86)		1.73 (0.92)		2.66 (0.94)		2.25 (0.78)	

**Table 2:** Mean Scores of Posttraumatic Growth, Wisdom, and Quality of Life

Variables	M (SD)
Posttraumatic growth (Range 0-5)	2.62 (0.92)
Spiritual change	2.08 (1.38)
New possibilities	2.37 (1.22)
Relating to others	2.74 (1.04)
Changed perception of self	2.77 (0.98)
Wisdom (Range 1-4)	2.90 (0.30)
Empathic emotion	3.05 (0.42)
Self-reflection	2.88 (0.31)
Bitter experience of life	2.69 (0.38)
Quality of life (Range 0-4)	2.47 (0.65)
Physical well-being	3.05 (0.86)
Social/family well-being	1.86 (0.92)
Emotional well-being	2.75 (0.87)
Functional well-being	2.25 (0.83)

**Table 3:** Correlations between Posttraumatic Growth, Wisdom and Quality of Life

Variables	Posttraumatic growth	Wisdom
	r (p)	r (p)
Posttraumatic growth	1	
Wisdom	0.57 (<0.001)	1
Quality of life		
Physical well-being	0.04 (0.692)	0.17 (0.066)
Social/family well-being	0.45 (<0.001)	0.42 (<0.001)
Emotional well-being	0.15 (0.093)	0.21 (0.024)
Functional well-being	0.27 (0.003)	0.40 (<0.001)

**Table 4:** Predictors on Each Domain of Quality of Life

Domain of QoL	Variable	B	SE	β	t (p)	F (p)	Adj R²
Physical well-being	SES	0.31	0.08	0.33	3.62 (<0.001)	6.14 (0.001)	0.136
Social/family well-being	SES	0.27	0.08	0.28	3.36 (0.001)	12.26 (<0.001)	0.348
	Wisdom	0.59	0.28	0.20	2.08 (0.040)		
	PTG	0.34	0.13	0.24	2.55 (0.012)		
Emotional well-being	SES	0.30	0.09	0.33	3.48 (0.001)	4.60 (<0.001)	0.195
Functional well-being	SES	0.17	0.08	0.19	2.14 (0.035)	6.66 (<0.001)	0.260
	Wisdom	0.79	0.28	0.29	2.87 (0.005)		

PTG,Posttraumatic growth; QoL,Quality of life; SES,Subjective economic status

Posttraumatic growth of cancer survivors is significantly associated with social/family QoL in this study. However, empirical evidence regarding the relationship between PTG and QoL following cancer diagnosis and treatments is inconclusive either a significant positive relationship (Tomich and Helgeson, 2012; Sim et al., 2014) or null or inverse relationship between them (Schwarzer et al., 2006; Thornton and Perez, 2006; Bellizzi et al., 2010). It is possible that inconsistencies in previous studies stem from no adequately capturing between PTG and each domain of QoL, because PTG may be associated with psychological or emotional QoL instead of physical or functional QoL as PTG co-occurs with distress and illness trauma (Bellizzi et al., 2010; Sim et al., 2014; Walsh et al., 2018). The greater the distress experienced by cancer survivors, the greater PTG they develop (Bellizzi et al., 2010). In line with this, the positive cognitive process that accompanies the experience of PTG leads to better psychological QoL (Sim et al., 2014). Therefore, psychological interventions to improve PTG would be needed to have a positive effect on older cancer survivors’ QoL.

Wisdom is associated with psychosocial outcomes such as positive values, enhanced mental health outcomes, and QoL (Ardelt, 1997; Staudinger and Glück, 2011; Jeste and Oswald, 2014). The wisdom of cancer survivors significantly affected functional and social QoL in this study. This is supported by previous studies that wisdom influences satisfaction with life, well-being and QoL (Ardelt, 1997, 2016; Webster et al., 2014). Facets of wisdom were formed by cognitive, reflective, and affective components (Ardelt, 2011). The cognitive component includes the ability to comprehend the deeper meaning of life events, and the reflective dimension consists of the ability to acquire different perspectives, overcome self-centeredness, and avoid blaming other individuals for one’s own circumstances (Ardelt, 2011). These facets of wisdom may influence on social/family and functional well-being, because people with high levels of wisdom-related performance had a higher degree of social relationships and functional well-being which involves experiences such as good interpersonal relationships, work engagement, and life satisfaction.

Several studies have demonstrated that the wisdom score increased with age, which suggests that wisdom is gained from an accumulation of diverse experiences in life (Kim and Min, 2010; Sung, 2011). However, the passage of time does not result in an increase of wisdom in everyone (Ardelt, 2016). Therefore, a program developed to aid an increase in wisdom would have a positive effect on QoL. Participants in the study were elderly, with an average age of 67.6 years, who survived cancer, and we speculate that the wisdom accumulated through life experiences, including traumatic experiences, would have positively influenced the functional and social QoL in these participants. Although the traumatic experience of cancer and aging may be viewed as negative experiences in the process of life, it is important to point it out that, through such processes, PTG can be experienced, wisdom can increase, and QoL can improve. This would help elderly cancer survivors have a positive perspective.

A strength of our study is that it addresses a significant gap in QoL research by examining relationships between PTG, wisdom and four domains of the QoL in older cancer survivors. A possible limitation of our study is that cancer survivors after completing treatments were drawn primarily from one public health center. Therefore, those participants may not represent the general population of older cancer survivors. Second, it is not possible to fully examine reciprocal relationships between variables because our study is cross sectional study design. Future study needs to include longitudinal data, which would be able to deeply understand the temporal relationship between PTG, wisdom and QoL.

In conclusion, the current study investigated the relationships between PTG, wisdom, and QoL in community dwelling older cancer survivors who had completed cancer treatments, and then to identify predictors of cancer survivors’ QoL. It is important to identify determinants of QoL among older cancer survivors, as it may provide potential interventions for improving their QoL. In this study, the most significant factor on each domain of QoL has shown that higher levels of SES were associated with significant improvement of four QoL domains, and wisdom and PTG were associated with significant improvement in social/family well-being. 

Given the paucity of research on community-dwelling older cancer survivors, there is a need for more research including exploratory and longitudinal studies. In particular, future study should be designed with longitudinal study design as cancer is now considered a chronic disease which affects individuals’ life over time. Furthermore, more research is needed on specific cancer survivor groups to evaluate their QoL. Finally, based on the study findings that PTG and wisdom significantly affected the QoL of cancer survivors, future study should be necessary to develop tailored interventions to improve their quality of life in community-dwelling cancer survivors. For example, either home-based cancer survivorship programs or survivorship wellness programs by a team of experts could help older cancer survivors learn about and practice to optimize their health and wellness after cancer treatment.
